# Effects of epidural anesthesia on the prognosis of ovarian cancer—a systematic review and meta-analysis

**DOI:** 10.1186/s12871-023-02352-1

**Published:** 2023-11-29

**Authors:** Haijian Shen, Qianyun Pang, Youzhu Gao, Hongliang Liu

**Affiliations:** Department of Anesthesiology, Chongqing University Cancer Hospital, Chongqing, China

**Keywords:** Ovarian neoplasm, Epidural anesthesia, Overall survival, Time to tumor recurrence

## Abstract

**Introduction:**

The global low survival rate among ovarian cancer patients has resulted in significant social and economic burdens. Nevertheless, previous studies have produced mixed results when exploring the link between anesthetic techniques and the prognosis of ovarian cancer. The study aims to compare the effect of epidural anesthesia with general anesthesia on survival time after cytoreductive surgery in patients with ovarian cancer.

**Methods:**

The PubMed (National Library of Medicine), Cochrane library, Web of science, Embase, CNKI (China National Knowledge Internet), Wanfang Med Online (China database), were systematically searched from inception to May, 2023, using the Medical Subject Headings [MeSH] of “Ovarian Neoplasm” and “Anesthesia, Epidural” and free words to identify systematic reviews or meta-analyses.

The research methodology involved analyzing randomized controlled trials (RCTs), as well as prospective or retrospective cohort studies, which compared the long-term prognosis of patients with ovarian cancer under general anesthesia combined with epidural anesthesia (GEA) versus general anesthesia alone (GA).

The Newcastle Ottawa Scale (NOS) was used to assess methodological quality and bias. Data extraction and assessment of study quality were conducted by two independent reviewers. A meta-analysis was then performed to calculate hazard ratios (HRs) and corresponding 95% confidence intervals (CIs). Overall survival (OS) was defined as the primary outcome, time to tumor recurrence (TTR) was the secondary outcome. Epidural anesthesia could be used intraoperatively and immediately postoperatively (EIP), or postoperatively only (EP). GEA includes EIP and EP.

**Results:**

In total, 8 retrospective cohort studies with 2036 participants met the inclusion criteria. The pooled results demonstrated that GEA could extend OS (HR 0.75, 95% CI 0.67–0.84, I^2^ = 0%, *P* < 0.05, fixed-effect model) when compared with GA in ovarian cancer patients undergoing cytoreductive surgery, but not TTR (sensitivity analysis revealed substantial heterogeneity among the included studies). The result of analyzing a total of 1490 patients in 4 studies was that EIP had a better prognosis on OS than GA (HR 0.68, 95%CI 0.55–0.85, I^2^ = 61%, *P* < 0.05, random-effect model). However, EP had no advantage in TTR (sensitivity analysis revealed it was unstable outcome). Ovarian cancer FIGO(International Federation of Gynecology and Obstetrics) stage III, stage IV compared to stage I on OS was statistically significant, HRs respectively are 3.67 (95%CI 2.25–5.98), I^2^ = 0%, fixed-effect model, *P* < 0.05, and 7.43 (95%CI 3.67–15.03), I^2^ = 31%, fixed-effect model, *P* < 0.05, but there was no statistically significant difference between stage II and stage I, HR 2.00, 95%CI0.98–4.09, I^2^ = 0%, fixed-effect model, *P* > 0.05. 1-10 mm tumor residuals shorten TTR compared with 0 residuals, HR 1.75, 95% CI1.50–2.04, I^2^ = 0%, fixed-effect model, *P* < 0.05.

**Conclusions:**

It is hard to conclude that postoperative epidural analgesia offers greater benefits than GA. However, general anesthesia combined with epidural anesthesia (EIP) can improve overall survival in ovarian cancer patients, allowing the anesthesiologist to use anesthesia techniques to provide a favorable prognosis for the ovarian cancer patient. Tumor staging and the extent of cell reduction are also critical factors that significantly influence the long-prognosis of ovarian cancer patients.

**Supplementary Information:**

The online version contains supplementary material available at 10.1186/s12871-023-02352-1.

## Other

The registration number in PROSPERO is CRD42021240831. We have refined the title of the article and expanded the range of included study types, because we were unable to find prospective randomized controlled trials. Additionally, we have modified the tool for assessing the risk of bias in the articles and instead utilized the Newcastle-Ottawa Scale (NOS) for evaluating cohort studies. A review protocol was not prepared for this study.

## Introduction

Ovarian cancer is a serious health concern worldwide, characterized by its aggressive nature and late-stage diagnosis. Ovarian cancer ranks as the fifth leading cause of cancer-related deaths among women worldwide [1]. In the United States, new cases of ovarian cancer were predicted to be around 19,880 in 2022, with 12,810 deaths. The overall 5-year survival rate remains dishearteningly low, with less than 50% of patients surviving beyond five years [2]. In China, the survival prospects for ovarian cancer are equally distressing. Majority of patients are diagnosed at advanced stages, resulting in poorer outcomes and limited treatment options [3]. Medical technology has progressed significantly in recent decades, but ovarian cancer remains the fatality leader among gynecological cancers. Due to the aggressiveness and ineffectiveness of treatment, ovarian cancer is rising in the incidence with a 5-year survival rate of less than 50% [4].

Primary cytoreductive surgery, followed by adjuvant chemotherapy is the standard treatment for ovarian cancer. However, surgery inhibits immune function. Retrospective investigations have revealed a link between anesthetic technique and cancer outcomes [5, 6], epidural anesthesia appears to exert an antitumorigenic action in cancer patients [7]. In fundamental research on RNA, epidural analgesia is linked to a decreased likelihood of ovarian cancer recurrence following initial cytoreductive surgery [8]. However, currently there is no systematic evaluation to validate this measure.

Accordingly, it is urgent to elucidate how anesthesia can influence the long-term outcomes of ovarian cancer. In light of these considerations, we conducted this meta-analysis to compare general anesthesia alone with general anesthesia combined with epidural anesthesia and tried to clarify the prognostic effectiveness of epidural anesthesia in patients with ovarian cancer.

## Materials and methods

The study aims to compare the effect of epidural anesthesia with general anesthesia on survival time after cytoreductive surgery in patients with ovarian cancer. We conducted this study following the 2020 PRISMA guidelines (Preferred Reporting Items for Systematic Reviews and Meta-Analysis). The protocol of this systematic review and meta-analysis was registered in PROSPERO, and the registration number is CRD42021240831. Funnel plots are the most commonly used visualizations to demonstrate publication bias. We used the funnel plot of the Review Manager (RevMan) 5.2 to observe publication bias. Sensitivity analysis was performed by excluding 1 document in turn and merging the remaining documents (n-1 documents) for meta-analysis, and assessing whether the results of the original meta-analysis were significantly altered by the influence of certain studies by observing the changes in the merged results. We performed subgroup analyses of the results depending on whether the administration of epidural anesthesia was intraoperative (EIP) or postoperative (EP) to test the stability of the results.

### Literature search

The PubMed (National Library of Medicine), Cochrane library, Embase, Web of Science, CNKI (China National Knowledge Internet), Wan fang Med Online were systematically searched from the inception dates to May, 2023, using the Medical Subject Headings [MeSH] of “Ovarian Neoplasm” and “Anesthesia, Epidural” and free words to identify systematic reviews or meta-analyses. Free words included “ovarian cancer”, “ovarian neoplasms”, “ovarian tumor”, “ovarian carcinoma”, “anesthesia, epidural”, “anesthesia, peridural”, “anesthesia, extradural”. Results of the database searches are displayed in the study flow diagram.

To discover additional or continuing studies, we reviewed the reference lists of relevant journals and contacted relevant trial authors. We also used the website ‘http://clinicaltrials.gov/’ to look for suitable trials. In June 2021, a computerized literature search was conducted with language restrictions (English and Chinese).

The literature search was carried out by SHJ and GYZ independently, with any conflicting viewpoints being assessed by PQY, a third party who assisted in reaching a consensus on the findings.

### Criteria for inclusion

We included publications in our meta-analysis if they met the following criteria: 1. they were independent prospective or retrospective cohort studies, RCTs; 2. the effect of combining epidural with general anesthesia on ovarian cancer outcome,1): overall survival (OS, the time elapsed between surgery and death from any cause) 2): time to tumor recurrence (TTR, the time elapsed between surgery and tumor recurrence), 3: studies provided enough useful data to calculate the hazard ratio (HR) with its 95% confidence intervals (CI). Reviews, meta-analyses and trials with insufficient data were excluded.

### Quality assessment

The Newcastle–Ottawa Quality Assessment Scale (NOS) was used to assess the study’s quality and bias potential. The NOS consists of eight items divided into three dimensions: selection, comparability, and outcome (cohort studies) or exposure (case–control studies), depending on the study type. Several response alternatives are presented for each issue. A star system is employed to allow for a semi-quantitative assessment of research quality, with the top-quality studies receiving a maximum of one star for each item, except for the comparability item, which receives two stars. The NOS is rated from one to nine stars. Receiving 7–9 stars is considered high-quality research [9]. Two review writers (SHJ and PQY) completed the “Risk of bias” based on this technique. We worked out our differences with the help of a third review author (LHL). In the results section, we provide the number of stars for each study.

### Data extraction

The data extraction was carried out independently by two qualified investigators (SHJ and GYZ). The initial author’s surname, the year of publication, design type, interventions, numbers in distinct groups, and outcomes were all collected in detail from each study. We selected Overall survival (OS) and time to tumor recurrence (TTR) as the primary outcome indicators. Multivariable Cox proportional hazards analysis of factors associated with prognosis from each article were compiled and summarized. The same prognostic factors of ovarian cancer listed in each article were selected for statistical analysis, and the statistical findings were used as secondary outcome.

### Statistical analysis

In this meta-analysis, the findings of eligible studies were pooled using Review Manager (RevMan) 5.2. The HR and the standard error were used to compare therapies for survival outcomes. HR was defined as an advantage for the intervention group and an advantage for the control group, with HR < 1 denoting an advantage for the intervention group and HR > 1 denoting an advantage for the control group. We made suitable adjustments to the HR calculated from individual trials. On a non-log scale, we report HRs with 95 percent confidence intervals (CIs). The general inverse variance approach with a fixed-effect model was used to estimate the summary HR across trials, using the statistical software RevMan if there was no heterogeneity among studies. It is considered heterogeneous if *P* < 0.05 or I^2^ > 50%. We also employed a random-effects model to analyze the data to address concerns about judging clinical heterogeneity. Subgroup analysis has been conducted to explore the sources of heterogeneity. Sensitivity analyses conducted to assess robustness of the synthesized results. *P* < 0.05 was defined as statistically significant.

## Results

**Fig. 1:**
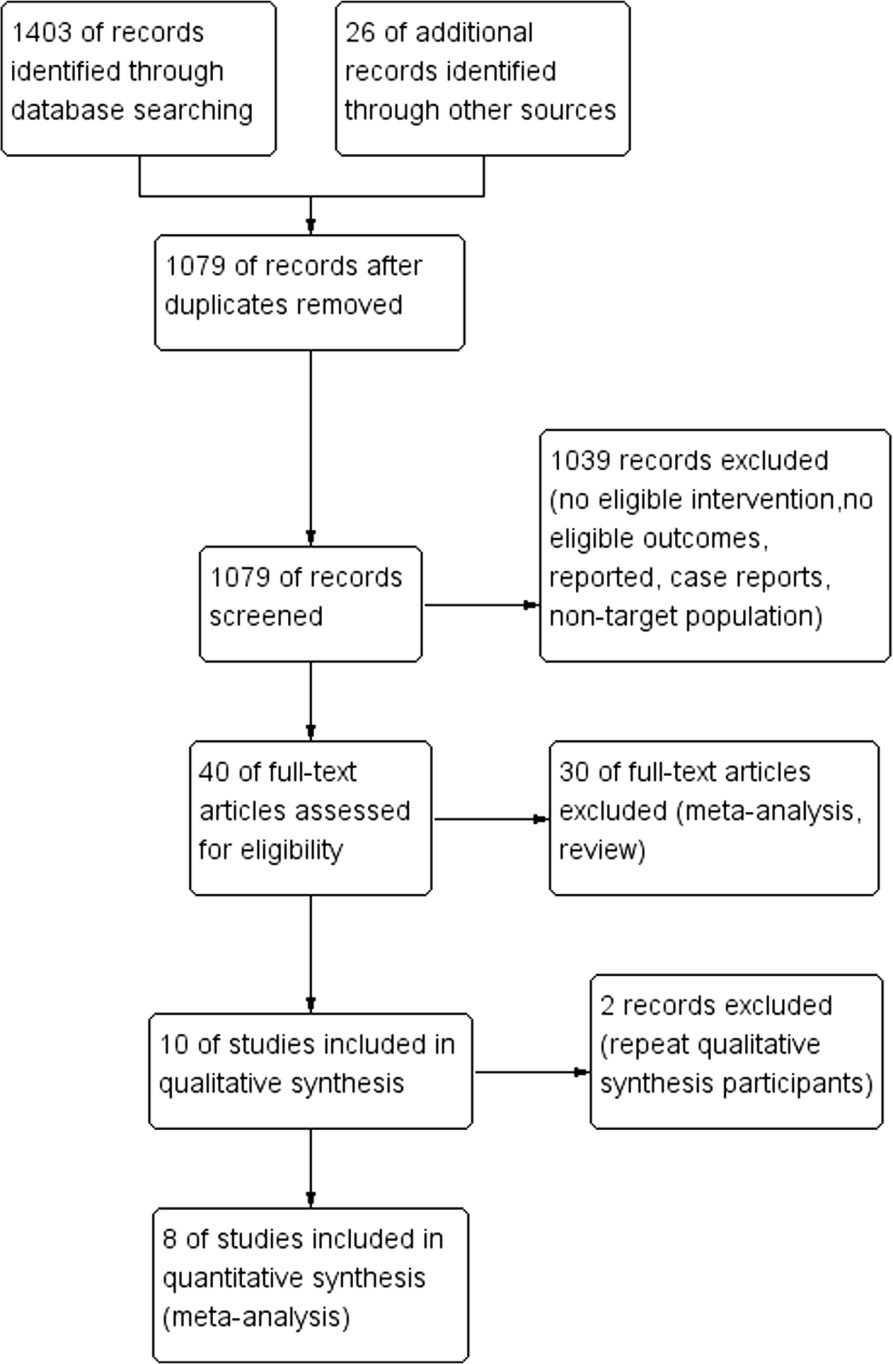
Flowchart of included and excluded studies

After the comprehensive search of the databases, we picked 8 studies [10–17] for our complete meta-analysis after reading the full text of all potentially eligible publications, including 948 cases in the general combined with epidural anesthesia (GEA, epidural anesthesia used postoperatively and/or intraoperatively) group and 1088 cases in the general anesthesia alone (GA) group. Postoperative intravenous analgesia was implemented in GA groups in these 8 researches. Figure 1 shows the flowchart for the literature search. The basic characteristics of studies are shown in Table 1. Intervention strategies included epidural anesthesia started either intraoperatively (EIP) or immediately postoperatively (EP).

**Table 1:** Basic characteristics of included studies

Authors	Study design	Interventions	Sample sizes (n)	Outcomes	Quality^a^
Anic K 2022	Retrospective Cohort	EIP VS GA	110	TTR, OS	9^a^
De Oliveira 2011	Retrospective Cohort	EIP VS GA EP VS GA	153	TTR	9^a^
Elias K.M 2015	Retrospective Cohort	EP VS GA	194	TTR	9^a^
Lacassie 2013	Retrospective Cohort	EIP or EP VS GA	55	TTR, OS	9^a^
Tseng 2018	Retrospective Cohort	EIP or EP VS GA	648	TTR, OS	8^a^
Capmas P 2012	Retrospective Cohort	EP VS GA	94	TTR, OS	8^a^
L Lin 2011	Retrospective Cohort	EIP VS GA	143	OS	9^a^
Huang 2018	Retrospective Cohort	EIP VS GA E VS GA	639	OS	9^a^

*EIP* General anesthesia combined with epidural anesthesia used not only intraoperatively but postoperatively, *EP* General anesthesia combined with epidural anesthesia used only postoperatively, *E* Epidural anesthesia only and without general anesthesia, *GA* General anesthesia with postoperative intravenous analgesia

^a^Evaluated by the 9-star Newcastle–Ottawa Scale

## Outcomes

### Quantitative data synthesis

#### Primary outcomes

##### Effect of GEA (EP and EIP) on OS

**Fig. 2:**
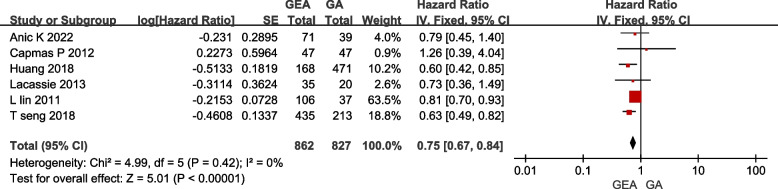
Effect GEA on OS

With a total of 1689 participants, we did a statistical analysis of 6 publications with OS as the primary outcome (outcomes of EIP interventions were chosen in Huang 2018), and got a result of HR 0.75, 95% CI 0.67–0.84, I^2^ = 0%, *P* < 0.05, using the fixed-effect model (Fig. 2). General anesthesia combined with epidural anesthesia used intraoperatively or postoperatively (EIP or EP), were found to be superior to general anesthesia with postoperative intravenous analgesia in terms of postoperative ovarian cancer overall survival time, with statistical significance.

**Subgroup analysis**

*Effect of EIP on OS*

**Fig. 3:**
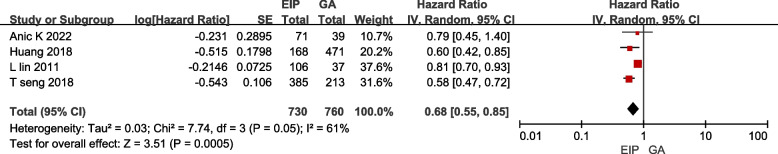
Effect of EIP on OS

Four papers, involving 1490 patients, compared EIP (general anesthesia combined with epidural anesthesia and analgesia) and GA (general anesthesia combined with intravenous analgesia) in terms of OS. The result (Fig. 3) shows that EIP is superior to GA on OS. HR 0.68, 95%CI 0.55–0.85, I^2^ = 61%, *P* < 0.05, using random-effect model.

#### Secondary outcomes

##### Effect of GEA (EP and EIP) on TTR

**Fig. 4:**
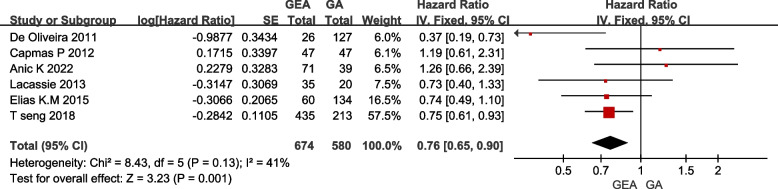
Effect of GEA on TTR

Six studies reported TTR in a total of 1254 individuals, and the result (Fig. 4) shows that general anesthesia combined with epidural anesthesia used intraoperatively or postoperatively could extend TTR when compared with general anesthesia with intravenous analgesia in ovarian cancer patients undergoing cytoreductive surgery (HR 0.76, 95% CI 0.65–0.90, I^2^ = 41%, *P* < 0.05, using fixed-effect model).

**Subgroup analysis**

*Effect of EP (epidural anesthesia used postoperatively) on TTR*

**Fig. 5:**
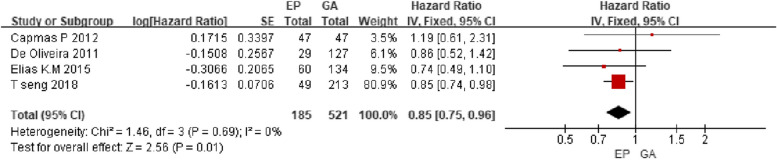
Effect of EP on TTR

A total of 706 patients were included in 4 literatures. Interventions were epidural anesthesia used postoperatively. That is to say, epidural analgesia was compared with intravenous analgesia on TTR in these 4 literatures. The statistical result (Fig. 5) shows that epidural analgesia has advantages on TTR compared with postoperative intravenous analgesia using fixed-effect model. HR 0.85, 95%CI 0.75–0.96, I^2^ = 0%, *P* < 0.05.

##### Effect of FIGO stage on OS

**Fig. 6:**
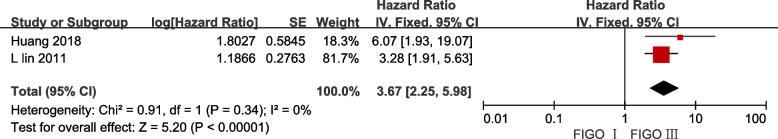
Effect of FIGO III VS FIGO I on OS

**Fig. 7:**
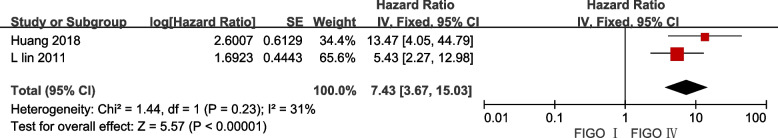
Effect of FIGO IV VS FIGO I on OS

**Fig. 8:**
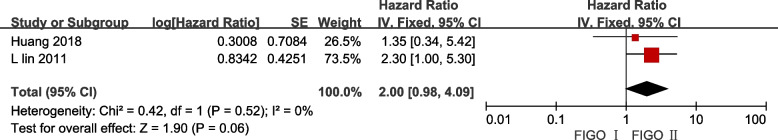
Effect of FIGO II VS FIGO I on OS

Ovarian cancer FIGO stage III (Fig. 6), stage IV (Fig. 7) compared to stage I on OS is statistically significant, as demonstrated in the figures below. HRs respectively are 3.67 (95%CI 2.25–5.98), I^2^ = 0%, using fixed-effect model, *P* < 0.05, and 7.43 (95%CI 3.67–15.03), I^2^ = 31%, fixed-effect model, *P* < 0.05. However, there is no statistically significant difference between stage II and stage I (Fig. 8), HR 2.00, 95%CI 0.98–4.09, I^2^ = 0%, fixed-effect model, *P* > 0.05.

##### Effect of the level of surgical debulking on TTR

**Fig. 9:**
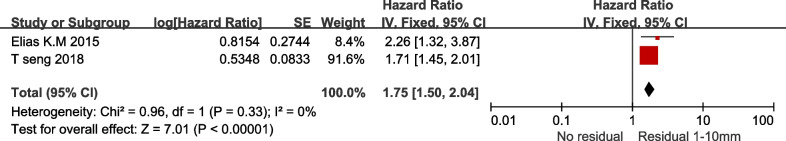
Effect of the level of surgical debulking on TTR

Two articles reported the effect of tumour residuals of 1-10 mm compared to 0 residuals in patients with ovarian cancer cell reduction surgery on TTR. We refered to other articles [18], pooled the HR of 1-5 mm residuals and 5-10 mm residuals listed in Elias K.M 2015, used the random-effects model, and then performed a statistical analysis. Results demonstrates that 1-10 mm tumor residuals shorten TTR compared with 0 residurals, HR 1.75, 95% CI 1.50–2.04, I^2^ = 0%, *P* < 0.05, using fixed-effect model (Fig. 9).

### Sensitivity analysis

The source of heterogeneity was assessed by means of a sensitivity analysis. We performed a sensitivity analysis on the main outcomes, revealing that the outcomes of GEA vs GA regarding TTR and EIP vs GA on TTR exhibited instability. By excluding studies with substantial weights, the results underwent a notable transformation from being statistically significant (*P* < 0.05) to becoming non-significant (*P* > 0.05). Nevertheless, the comparison outcome for OS remained consistently stable, the pooled HRs were not affected by any single study, as shown in the tables (Tables 2 and 3) provided below for further details, indicating that the results of our meta-analysis are robust and stable. A comprehensive examination of these findings will be presented in the subsequent discussion section.

**Table 2:** Effect of GEA on OS

Excluded Study	HR (95%CI)	*P* Value
Anic 2022	0.75(0.66,0.84)	< 0.05
Capmas P 2012	0.74(0.66,0.83)	< 0.05
Huang 2018	0.77(0.68,0.86)	< 0.05
Lacassie 2013	0.75(0.67,0.84)	< 0.05
L Lin 2011	0.66(0.64,0.79)	< 0.05
Tseng 2018	0.78(0.69,0.88)	< 0.05

**Table 3:** Effect of EIP on OS

Excluded Study	HR (95%CI)	*P* Value
Anic 2022	0.67(0.52,0.86)	< 0.05
Huang 2018	0.71(0.55,0.92)	< 0.05
L Lin 2011	0.60(0.51,0.71)	< 0.05
Tseng 2018	0.77(0.68,0.88)	< 0.05

### Publication bias

**Fig. 10:**
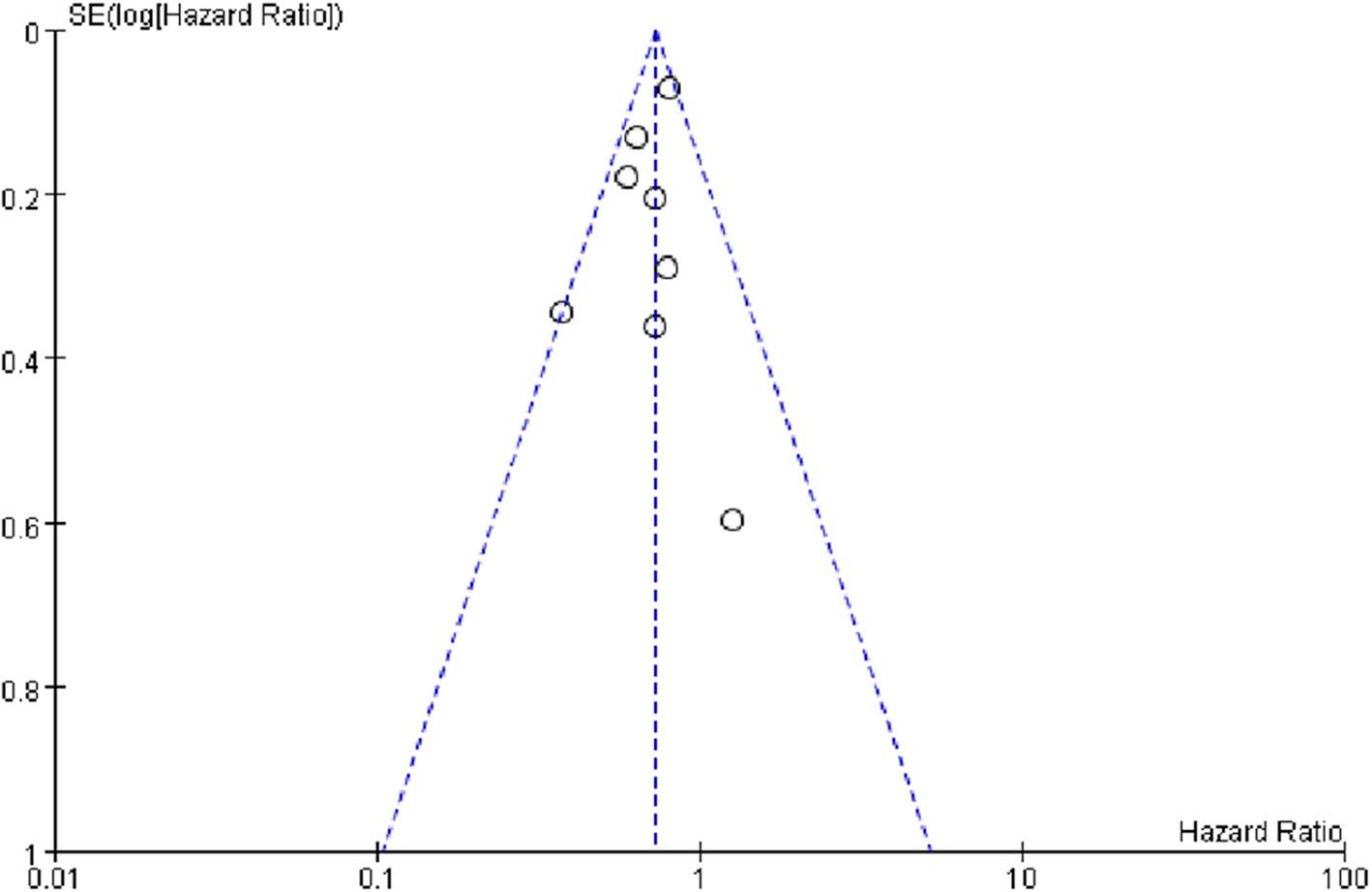
The funnel plot showed no significant publication bias

We used Revman 5.0 to draw funnel plots to assess publication bias for this meta-analysis (Fig. 10). In the funnel plot, the horizontal axis represents the effect size (HR) and the vertical axis represents the inverse of the precision of the study (standard error). Each point represents an independent study and its position reflects the effect size and precision of the study. Upon examining the distribution of the funnel plot, it was observed that the data points demonstrated a predominantly symmetrical inverted funnel shape, suggesting a limited probability of publication bias. Based on these findings, it can be concluded that the meta-analysis exhibited a low level of publication bias, rendering the obtained results reasonably reliable.

## Discussion

The meta-analysis showed an association between GEA and ovarian cancer patients’ overall survival. The same result was found in the subgroup analysis of EIP. Our results from the comprehensive meta-analysis coincided with most, even not all, results from recently reported studies [5, 19]. According to the COX regression analysis of included studies, the long-term prognosis of ovarian cancer patients after surgery was also related to tumor stage, whether the tumor reduction operation was complete, as showed up the outcomes part. The results of these studies are crucial in future prospective studies investigating the intriguing prospect of appropriate perioperative GEA modifying cancer recurrence.

However, the outcomes of GEA vs GA regarding TTR and EIP vs GA on TTR exhibited instability. Epidural anesthesia as an intervention shows advantages in improving overall survival (OS) in ovarian cancer patients, whereas its effect on improving TTR remains unclear, similar to the impact of combined epidural-general anesthesia on long-term survival in colorectal cancer and other kinds of cancer patients [20–22]. The reason for this discrepancy could be attributed to the fact that overall survival has a clearly defined time point, whereas TTR depends on the timing of patient follow-up visits, making it difficult to precisely define. Moreover, the definition of TTR is not standardized. The appearance of new lesions on imaging or an increase in CA125 levels can both be used as endpoints for defining TTR. However, the magnitude of the increase in CA125 levels is uncertain, and there is also inconsistency in imaging resolution and the diagnostic proficiency of physicians. Furthermore, all articles are retrospective ones, and most studies did not perform propensity score matching when comparing two groups. Compared to randomized controlled studies, they lack strong credibility, which is also a reason for the instability of the results. Consequently, when pooling results using TTR as an outcome, the stability of the outcome becomes uncertain.

Two articles included in this study, Lacassie 2013 and Tseng 2018, both mixed the two interventions of EIP and EP for statistical analysis, but the results were not consistent. Comparison of the two interventions (Table 4): epidural analgesic was 0.1–0.5% bupivacaine with or without opioids in Lacassie 2013, duration was “All epidural catheters remained in place for at least 48 h”. The article did not mention the respective proportions of epidural anesthesia and analgesia; the epidural analgesic in Tseng 2018 was 0.05% bupivacaine, with or without opioids, and most patients can receive oral analgesia, epidural analgesia was discontinued on medication for up to 14 days, and the rate of epidural anesthesia was more than epidural analgesia that of started postoperatively (89% vs 11%). Study populations were similar (Lacassie 2013: stage IIIC and IV, Tseng 2018: stage IIIB and IV). In conclusion, it appears that the reason Tseng 2018 obtained positive results is that there was a higher proportion of participants who received EIP compared to those who received EP, and the duration of epidural analgesia was longer. This result suggests that intraoperative epidural medication, which reduces intraoperative stress and intraoperative opioid consumption, may be beneficial to the survival prognosis of such patients, but not epidural analgesia. It is consistent with other studies [23, 24].

**Table 4:** Differences between Lacassie 2013 and Tseng 2018

	Lacassie 2013	Tseng 2018
Interventions	General anesthesia combined with epidural anesthesia used intraoperatively or postoperatively	
Medications	0.1–0.5% bupivacaine with or without opioids	0.05% bupivacaine, with or without opioids
Duration	At least 48 h	Up to 14 days
Proportion	/	intraoperatively VS postoperatively: 89% VS 11%
Outcomes	OS:HR 0.74(95%CI 0.36, 1.49) TTR:HR 0.73(95%CI 0.40, 1.31)	OS:HR 0.63(95%CI 0.49, 0.82) TTR:HR 0.75(95%CI 0.61,0.93)
Study populations	Stage IIIC and IV	Stage IIIB and IV

In general, epidural anesthesia has positively effect on the prognosis of ovarian cancer patients after cytoreductive surgery. We all know that both surgery and anesthetics may suppress the body’s immune system, causing cancer to grow and spread. The mechanisms might be explained as follows. Initially, the body’s immune function can be inhibited by opioid analgesics in a dose-dependent manner, and affect both cellular and humoral immunity when they act directly on immune cells, the hypothalamic-pituitary–gonadal axis and sympathetic activity [25], especially on NK cell activity. Morphine, alfentanil, remifentanil, fentanyl and sufentanil not only suppress NK cell activity, but change T cell differentiation. Opioids usually inhibit T-lymphocyte proliferation [26]. Furthermore, investigations have demonstrated that opioid analgesics can promote tumor angiogenesis, which can drive tumor growth and invasion [27]. Volatile anesthetics also affect immune response. Halothane decreases NK cell activity and increases expression of hypoxia-inducible factor 1α (HIF-1α) [28, 29], and sevoflurane induces T-lymphocyte apoptosis and upregulates HIF-1α expression [29, 30]. Isoflurane, sevoflurane, and desflurane have also been demonstrated to up-regulate vascular endothelial growth factor A (VEGF-A), matrix metalloproteinase 11(MMP11), transforming growth factor 1 (TGF-1) and chemotaxis in investigations. The role of chemokine receptor-2 (CXCR-2) and other cell signal transduction and protein expression related to tumor metastasis directly promotes the metastasis of ovarian cancer tumor cells [31]. However, local anesthetics play a positive role. For example, ropivacaine restrained ovarian cancer cell stemness and accelerated cell ferroptosis by inactivating PI3K/AKT signaling pathway [32]. Epidural anesthesia reduces the dosage of opioid drugs and inhalational anesthetics, thereby minimizing their negative effects while also exerting a beneficial effect in inhibiting tumor recurrence. In summary, these findings broadly support the association between epidural anesthesia and the longer survival of ovarian cancer patients.

Therefore, we boldly predict that whether the use of intrathecal (IT) infusion of local anesthetic, like bupivacaine, to treat patients with chronic refractory pain has fewer adverse reactions and prolongs the survival time compared with opioid drugs such as morphine alone. Dose escalation with chronic IT opiates has been a cause for concern [33]. Research has demonstrated that local anesthetics, such as bupivacaine, exhibit a synergistic interaction with opiates [34]. From a logical standpoint, it is believed that effective pain management positively contributes to extending the survival time of patients. However, at present, there is a lack of research evidence to support this claim. Further research is needed in this area.

Of course, there are different meta-analysis conclusions. Epidural anesthesia combined with general anesthesia reduces tumor recurrence and metastasis in patients with prostate cancer but not with colorectal cancer [35]. The above findings may be attributed to epidural anesthesia having different effects on different tumors, which may be related to different pathologies and metastases. Another factor that we discussed for this effect was the duration of surgery. Ovarian cancer cytoreductive surgery often requires joint operation of several departments, the operation scope is large and time-consuming. There may be a difference between patients undergoing epidural anesthesia for colorectal cancer and prostate cancer due to the longer operative time for the latter [36, 37]. Therefore, we believe that epidural anesthesia’s effect is statistically significant only for time-consuming and traumatic operations. However, large-scale clinical studies involving different cancer types are needed to investigate the potential influence of the anesthetic technique used during surgery on cancer-related outcomes.

The tumor stage in ovarian cancer patients has a significant impact on prognosis [38]. Higher stages of ovarian cancer (such as stage III and IV) are associated with a worse prognosis compared to lower stages (stage I and II). Higher stages indicate more extensive spread of the tumor beyond the ovaries, which makes treatment more challenging and increases the risk of recurrence.

Regarding the impact of residual nodules during cytoreductive surgery for ovarian cancer cells, it has been observed that smaller residual nodules are associated with longer recurrence-free intervals and improved overall survival rates [39]. This suggests that achieving complete tumor removal during surgery is crucial for better outcomes.

However, our conclusions should also be interpreted carefully, as more randomized controlled trials are needed to verify them.

### Limitations

All the cited articles omitted any mention of total opioid use, thereby rendering it arduous to quantify the impact of opioids, which hold a significant role in general anesthesia. The effect of inhalation anesthetics on the prognosis of ovarian cancer is not discussed in detail in this study.

The studies incorporated in this analysis were all retrospective and not randomized controlled trials. Although we employed the Newcastle–Ottawa Scale (NOS) to assess the potential bias within these cohort studies, yielding outcomes of high quality, there still exist uncontrollable variables, such as substantial discrepancies in the number of research participants between groups. Anesthesiologists exhibited a preference for epidural anesthesia in patients suitable for complete debulking, which inevitably influenced the outcomes. Furthermore, the FIGO stage of patients in each study exhibited inconsistency, consequently impacting the survival time.

Despite the absence of RCTs among the articles encompassed in this meta-analysis, the intervention of epidural anesthesia and analgesia, after accounting for numerous confounding factors, still merits recommendation and support. We eagerly await large-scale randomized controlled trials that will furnish further elucidation.

## Conclusions

The current scenario of ovarian cancer survival and treatment paints a bleak picture both globally and domestically. Late-stage diagnosis, limited surgical options, and a lack of effective therapies contribute to the grim prognosis. However, epidural anesthesia during surgery can reduce stress response and enhance survival in ovarian cancer patients, allowing the anesthesiologist to use anesthesia techniques to provide a favorable prognosis for the ovarian cancer patient.

It is difficult to encapsulate the benefit of single postoperative epidural analgesia from each research review, as compared to general anesthesia with postoperative intravenous analgesia.

### Supplementary Information

**Additional file 1: Supplementary Table 1.** Listing the research methods, participants, interventions, outcomes, notes, scores of Newcastle-Ottawa Scale and support for judgement for each study- Characteristics of included studies.

**Additional file 2: Supplementary Table 2.** The summary of the HRs and 95% CI obtained from COX regression models for ovarian cancer patients as reported in the 8 studies-Summary of COX regression analysis.

**Additional file 3: Supplementary Table 3.** Including patients number of GEA and GA, EIP or EP and adminstered medicine, intravenous analgesia medicine, postoperative pain scores- Detailed comparison of GEA and GA.

## Data Availability

All data generated or analyzed during this study are included in this published article.

## Abbreviations

CI: Confidence interval
CNKI: China National Knowledge Internet
EIP: Epidural anesthesia used intraoperatively and postoperatively
EP: Epidural anesthesia used only postoperatively
FIGO: International Federation of Gynecology and Obstetrics
GA: General anesthesia
GEA: General anesthesia combined with epidural anesthesia
HR: Hazard ratio
MeSH: Medical Subject Heading
NOS: Newcastle-Ottawa Quality Assessment Scale
OS: Overall survival
PRISMA: Preferred Reporting Items for Systematic Reviews and Meta-Analysis
RCT: Randomized controlled trial
RevMan: Review Manager
TTR: Time to tumor recurrence
