# Supplementary material for: Effects of epidural anesthesia on the prognosis of ovarian cancer—a systematic review and meta-analysis
Source: BMC Anesthesiol. 2023 Nov 29;23:390. doi: 10.1186/s12871-023-02352-1 (PMC10685604; doi:10.1186/s12871-023-02352-1)
Supplement: Supplementary file 1 — Additional file 1: Supplementary Table 1. Listing the research methods, participants, interventions, outcomes, notes, scores of Newcastle-Ottawa Scale and support for judgement for each study- Characteristics of included studies. [file 12871_2023_2352_MOESM1_ESM.doc]

**Characteristics of included studies**

| **Capmas P 2012** | | | |
| --- | --- | --- | --- |
| Methods | | retrospective analysis | |
| Participants | | women who had undergone complete cytoreduction for ASOC between January 2007 and December 2009 in a single cancer reference center. Patients in whom cytoreduction had been incomplete or patients with a nonepithelial ovarian tumor were excluded, as were these who had been treated for recurrent disease. | |
| Interventions | | Patient-controlled epidural analgesia (PCEA) | |
| Outcomes | | overall survival and recurrence-free survival | |
| Notes | | One hundred and four women were included in the study. Fifty-one women had undergone epidural analgesia while 53 had not. Ten women had been lost to follow-up, four in the PCEA group (8%) and six in the no-PCEA group (11%). Thus, the analysis was performed on 47 women in each group | |
| ***Risk of bias 8**** | | | |
| **Bias** | | **Authors’ judgement** | **Support for judgement** |
| Selection | Representativeness of the exposed cohort | *truly representative of the average advanced-stage ovarian cancer women (describe) in the community | This retrospective analysis included women who had undergone complete cytoreduction for ASOC between January 2007 and December 2009 in a single cancer reference center. |
|  | Selection of the non exposed cohort | *drawn from the same community as the exposed cohort |  |
|  | Ascertainment of exposure | *secure record (e.g., surgical records) | Patients’ medical records were retrospectively reviewed to extract  demographic, perioperative and follow-up data. Age, tumor  histology and grade were extracted from patients’ charts. |
|  | Demonstration that outcome of interest was not present at start of study | *yes | Outcomes need to be obtained from follow-up |
| Comparability | Comparability of cohorts on the basis of the design or analysis | *study controls for who had received PCEA | Patient-controlled epidural analgesia (PCEA) was often proposed for postoperative analgesia, in the absence of contraindications, especially when cytoreduction was expected to be wide or to include a digestive resection. |
|  |  | *study controls for any additional factor | After verification of proportionality of the hazard ratio, potential prognostic factors for recurrence-free and overall survival were first assessed by univariate analysis using a Cox regression model. |
| Outcome | Assessment of outcome | *record linkage |  |
|  | Was follow-up long enough for outcomes to occur | Not select an adequate follow up period for outcome of interest | / |
|  | Adequacy of follow up of cohorts | *description provided of those lost | Ten women had been lost to follow-up, four in the PCEA group (8%) and six in the no-PCEA group (11%).  Thus, the analysis was performed on 47 women in each group. |

| **De Oliveir 2011** | | | |
| --- | --- | --- | --- |
| Methods | | retrospective analysis | |
| Participants | | patients with a diagnosis of primary epithelial ovarian cancer who had undergone initial cytoreductive surgery at Prentice Women’s Hospital between January 2000 and October 2006 were identified from the gynecology-oncology database using current procedural terminology codes. | |
| Interventions | | Patient-controlled epidural analgesia (PCEA) and epidural anesthesia | |
| Outcomes | | time to tumor recurrence and survival | |
| Notes | | The medical records of 232 patients with a diagnosis of ovarian cancer who underwent optimal cytoreductive surgery between January 2000 and October 2006 and who met inclusion criteria were reviewed. Fifty subjects were excluded from the analysis for the following reasons: 28 were benign or borderline tumors, 7 were secondary cytoreductive surgery, 8 were primary peritoneal tumors, 2 were germ cell tumors, 2 had primary uterine origin, 2 were not optimally ‘‘debulked,’’ and 1 was a  primary fallopian tube carcinoma. The median (interquartile range [IQR]) follow-up time was 42 (12Y60) months. | |
| ***Risk of bias 9**** | | | |
| **Bias** | | **Authors’ judgement** | **Support for judgement** |
| Selection | Representativeness of the exposed cohort | *truly representative of the average ovarian cancer women (describe) in the community | patients with a diagnosis of primary epithelial ovarian cancer who had undergone initial cytoreductive surgery at Prentice Women’s Hospital between January 2000 and  October 2006 was identified from the gynecology-oncology database using current procedural terminology codes |
|  | Selection of the non exposed cohort | *drawn from the same community as the exposed cohort |  |
|  | Ascertainment of exposure | *secure record (e.g., surgical records) ¯ | extracted from patient’s medical records |
|  | Demonstration that outcome of interest was not present at start of study | *yes | Outcomes need to be obtained from follow-up |
| Comparability | Comparability of cohorts on the basis of the design or analysis | *study controls for who had received PCEA or epidural anesthesia (select the most important factor) | Subject characteristics were compared among subjects who did not receive epidural anesthesia, those who had epidural anesthesia intraoperatively as well as postoperatively, and those who received epidural analgesia postoperatively only. |
|  |  | *study controls for any additional factor - (These criteria could be modified to indicate specific control for a second important factor.) | A Cox proportional hazard regression model was constructed to adjust for confounding variables for time to tumor recurrence |
| Outcome | Assessment of outcome | * record linkage | medical records |
|  | Was follow-up long enough for outcomes to occur | *yes (select an adequate follow up period for outcome of interest) | Follow-up assessments were made at 3-month intervals for the first and second years, 6-month intervals during the third  and fourth years, and every 12 months beginning the fifth year after the surgery. |
|  | Adequacy of follow up of cohorts | *description provided of those lost | Fifty subjects were excluded from the  analysis for the following reasons: 28 were benign or borderline tumors, 7 were secondary cytoreductive surgery, 8 were primary peritoneal tumors, 2 were germ cell tumors, 2 had primary uterine origin, 2 were not optimally ‘‘debulked,’’ and 1 was a primary fallopian tube carcinoma. |

| **Elias K.M 2015** | | | |
| --- | --- | --- | --- |
| Methods | | historical cohort study | |
| Participants | | Women with Stage III epithelial ovarian cancer treated at Brigham and Women’s Hospital from January 1, 2007, until December 31,2011, were reviewed. | |
| Interventions | | Epidural analgesia and volatile anesthetics | |
| Outcomes | | DFS (disease-free survival) | |
| Notes | | Between January 1, 2007, and December 31, 2011, 194 cases met study inclusion criteria. The median age was 61 years. Most patients were parous, non-Hispanic, white women. | |
| ***Risk of bias 9**** | | | |
| **Bias** | | **Authors’ judgement** | **Support for judgement** |
| Selection | Representativeness of the exposed cohort | *truly representative of the average ovarian cancer women (describe) in the community | All cases of primary debulking surgery for ovarian cancer treated via exploratory  laparotomy from January 1, 2007, until December 31,2011, were reviewed. |
|  | Selection of the non exposed cohort | *drawn from the same community as the exposed cohort |  |
|  | Ascertainment of exposure | *secure record (e.g., surgical records) | extracted from patient’s medical records |
|  | Demonstration that outcome of interest was not present at start of study | *yes | Outcomes need to be obtained from follow-up |
| Comparability | Comparability of cohorts on the basis of the design or analysis | *study controls for who had received PCEA and volatile anesthetics | We assess the effects of epidural analgesia and volatile anesthetics as independent primary exposures |
|  |  | *study controls for any additional factor - (These criteria could be modified to indicate specific control for a second important factor.) | and then use a multivariate analysis to discuss the potential for an optimal anesthetic regimen for ovarian cancer debulking. |
| Outcome | Assessment of outcome | * record linkage | Deidentified study data were collected and managed on local secure servers using REDcap electronic data capture tools |
|  | Was follow-up long enough for outcomes to occur | *yes (select an adequate follow up period for outcome of interest) | All patients had at least 2 years of follow-up data available for review, with a median follow-up of 46 months (interquartile interval 31–68 months). |
|  | Adequacy of follow up of cohorts | *complete follow up - all subjects accounted for | All patients had at least 2 years of follow-up data available for review |

| **Huang 2018** | | | |
| --- | --- | --- | --- |
| Methods | | Retrospective analysis | |
| Participants | | Between January 2006 and December 2010, 976 patients who underwent ovarian cancer cell reduction surgery at Hunan Cancer Hospital were chosen, and the data of 903 patients was included in the research based on the exclusion criteria below. | |
| Interventions | | Spinal anesthesia, general anesthesia combined with epidural anesthesia | |
| Outcomes | | Overall survival | |
| Notes | | The research involved a total of 903 patients. 68 individuals were lost to follow-up or had medical data that were insufficient. Two of the patients had additional tumors, and three of them died of other conditions. | |
| ***Risk of bias 9**** | | | |
| **Bias** | | **Authors’ judgement** | **Support for judgement** |
| Selection | Representativeness of the exposed cohort | *truly representative of the average ovarian cancer women (describe) in the community | 976 patients between January 2006 and December 2010, who underwent ovarian cancer cell reduction surgery at Hunan Cancer Hospital |
|  | Selection of the non exposed cohort | *drawn from the same community as the exposed cohort |  |
|  | Ascertainment of exposure | *secure record (e.g., surgical records) | The data were obtained from electronic medical record system and follow-up system of Hunan Cancer Hospital |
|  | Demonstration that outcome of interest was not present at start of study | *yes | Outcomes need to be obtained from follow-up |
| Comparability | Comparability of cohorts on the basis of the design or analysis | *study controls for who had received epidural anesthesia and analgesia with or without general anesthesia | There are three types of anesthesia: general anesthesia, spinal anesthesia and general anesthesia combined with epidural anesthesia |
|  |  | *study controls for any additional factor - (These criteria could be modified to indicate specific control for a second important factor.) | Univariate COX regression was used to investigate the influence of baseline or intraoperative variables on overall survival (OS). |
| Outcome | Assessment of outcome | * record linkage | Patients' basic information, admission records, examination results, medical advice records, diagnosis and treatment plans, diagnosis records, anesthetic records, surgery records, and other information are all stored in an electronic medical record system. |
|  | Was follow-up long enough for outcomes to occur | *yes (select an adequate follow up period for outcome of interest) | Finally, patients were tracked for five years following surgery or until they died. The study's data collecting finished in December 2015. |
|  | Adequacy of follow up of cohorts | *description provided of those lost | 68 patients were lost to follow-up or had medical data that were insufficient. Two of the cancers were compounded by additional tumors, and three of the patients died of other conditions. |

| **Lacassie 2013** | | | |
| --- | --- | --- | --- |
| Methods | | Retrospective analysis | |
| Participants | | The anesthesia records of all de novo FIGO stage IIIC and IV cases undergoing surgery for ovarian cancer between January 2000 and March 2011 were reviewed. | |
| Interventions | | Epidural anesthesia and/or analgesia (EA) | |
| Outcomes | | Time to recurrence and overall survival | |
| Notes | | the median time to recurrence was 1.6 and 1.4 years for the EA and no EA groups, respectively (P = 0.30), median survival time was 3.3 and 2.7 years for the EA and no EA groups, respectively (P = 0.37) | |
| ***Risk of bias 9**** | | | |
| **Bias** | | **Authors’ judgement** | **Support for judgement** |
| Selection | Representativeness of the exposed cohort | *truly representative of the average ovarian cancer women (describe) in the community | The anesthesia records of all de novo FIGO stage IIIC and IV cases undergoing surgery for ovarian cancer between January 2000 and March 2011 were reviewed. |
|  | Selection of the non exposed cohort | *drawn from the same community as the exposed cohort |  |
|  | Ascertainment of exposure | *secure record (e.g., surgical records) | Patients were identified from a prospective clinical registry maintained by the Division of  Gynecologic Oncology under care of J. Brañes, MD |
|  | Demonstration that outcome of interest was not present at start of study | *yes | Outcomes need to be obtained from follow-up |
| Comparability | Comparability of cohorts on the basis of the design or analysis | *study controls for who had received epidural anesthesia and/or analgesia | Differences in baseline characteristics between patients who received epidural anesthesia and/or analgesia (EA) and those who did not receive EA (group no EA) were evaluated |
|  |  | *study controls for any additional factor - (These criteria could be modified to indicate specific control for a second important factor.) | We then fit a weighted Cox proportional hazards model using an indicator variable representing EA status as the sole predictor. |
| Outcome | Assessment of outcome | * record linkage | Patients were identified from a prospective clinical registry maintained by the Division of  Gynecologic Oncology under care of J. Brañes, MD |
|  | Was follow-up long enough for outcomes to occur | *yes (select an adequate follow up period for outcome of interest) | Follow-up assessments were conducted  at 3-month intervals for the first 2 years, followed by assessments every 6 months for 3 years, and then annually. |
|  | Adequacy of follow up of cohorts | *description provided of those lost | Nine patients were excluded from the analysis due to incomplete documentation of the anesthesia technique. The median follow-up time was 4.9 (interquartile  range: 2.5–9.8) years. |

| **L lin 2011** | | | |
| --- | --- | --- | --- |
| Methods | | Retrospective analysis | |
| Participants | | The medical records of all patients who underwent ovarian serous adenocarcinoma surgery at the center between January 1994 and October 2006, were reviewed. Only the records from newly diagnosed patients were included. Patients who underwent surgery more than once were excluded. | |
| Interventions | | Epidural anesthesia and analgesia | |
| Outcomes | | Survival time | |
| Notes | | The 1-, 3-, and 5-yr overall survival rates were 96% (95% CI, 92 –99%), 78% (95% CI, 70 –86%), and 61% (95% CI, 52–71%) in Group E, whereas 78% (95% CI, 64–91%), 58% (95% CI, 42–74%), and 49% (95% CI, 32 –65%) in Group G, respectively | |
| ***Risk of bias 9**** | | | |
| **Bias** | | **Authors’ judgement** | **Support for judgement** |
| Selection | Representativeness of the exposed cohort | *truly representative of the average ovarian cancer women (describe) in the community | The medical records of all patients who underwent ovarian serous adenocarcinoma surgery at the Centre, between January 1994 and October 2006, were reviewed. |
|  | Selection of the non exposed cohort | *drawn from the same community as the exposed cohort |  |
|  | Ascertainment of exposure | *secure record (e.g., surgical records) | medical records |
|  | Demonstration that outcome of interest was not present at start of study | *yes | Outcomes need to be obtained from follow-up |
| Comparability | Comparability of cohorts on the basis of the design or analysis | *study controls for who had received epidural anesthesia and analgesia | Group E included patients who underwent surgery with epidural anesthesia and analgesia, and Group G included patients who had GA combined with postoperative i.v. fentanyl analgesia. |
|  |  | *study controls for any additional factor - (These criteria could be modified to indicate specific control for a second important factor.) | For the primary analysis, we compared the survival rate using multivariable Cox’s proportional hazards regression while adjusting for any baseline or intraoperative factors independently related with the outcome |
| Outcome | Assessment of outcome | * record linkage | medical records |
|  | Was follow-up long enough for outcomes to occur | *yes (select an adequate follow up period for outcome of interest) | The evaluation cut-off time was November 2008 |
|  | Adequacy of follow up of cohorts | *description provided of those lost | 35 were excluded in Group G and  56 were excluded in Group E |

| **Tseng 2018** | | | |
| --- | --- | --- | --- |
| Methods | | Retrospective cohort study | |
| Participants | | We identified all patients diagnosed with stage IIIB to IV, high-grade epithelial ovarian, fallopian tube, or primary peritoneal cancer who had undergone PDS at our institution between January 1, 2005 and December 31, 2013. | |
| Interventions | | Epidural anesthesia and/or analgesia | |
| Outcomes | | Progression-free survival (PFS), Overall survival (OS) | |
| Notes | | Epidural group Median PFS 20.8 months OS 62.4 months;  Non-epidural group PFS 13.9 months OS 41.9 months (P<0.05) | |
| ***Risk of bias 8**** | | | |
| **Bias** | | **Authors’ judgement** | **Support for judgement** |
| Selection | Representativeness of the exposed cohort | *truly representative of the average ovarian cancer women (describe) in the community | We identified all patients diagnosed with stage IIIB to IV, high-grade epithelial ovarian, fallopian tube, or primary peritoneal cancer who had undergone PDS at our institution between January 1, 2005 and December 31, 2013 |
|  | Selection of the non exposed cohort | *drawn from the same community as the exposed cohort |  |
|  | Ascertainment of exposure | *secure record (e.g., surgical records) | Patients were only included if there was clear documentation of whether or not  epidural anesthesia was used. |
|  | Demonstration that outcome of interest was not present at start of study | *yes | Outcomes need to be obtained from follow-up |
| Comparability | Comparability of cohorts on the basis of the design or analysis | *study controls for who had received epidural anesthesia and/or analgesia | Epidural anesthesia was started intraoperatively in 385 (89%) and immediately postoperatively in 49 (11%). |
|  |  | *study controls for any additional factor - (These criteria could be modified to indicate specific control for a second important factor.) | Multivariable Cox PH models were used to examine variables independently associated with survival outcomes. |
| Outcome | Assessment of outcome | * record linkage | Medical records |
|  | Was follow-up long enough for outcomes to occur | Not given the last follow up time | / |
|  | Adequacy of follow up of cohorts | *description provided of those lost | Seventy-six patients were excluded  due to a lack of documentation regarding the presence or absence of epidural use |

| **Anic 2022** | | | |
| --- | --- | --- | --- |
| Methods | | Retrospective cohort study | |
| Participants | | Patients with all FIGO stages of ovarian cancer older than 60 years of age, who underwent standardized surgical treatment at the University Medical Center of the Johannes Gutenberg University Mainz between January 2008 and December 2019, were included. | |
| Interventions | | Perioperative epidural anesthesia | |
| Outcomes | | Progression-free survival (PFS), Overall survival (OS) | |
| Notes | | The median survival time for all OC patients was 26.0 months (11.8‑38.0). The ‘Non‑Epidural’ cohort survived with 27.0 months (15.0‑39.0) longer than the‘Epidural’‑supplemented group with 19.0 months (8.0‑36.5) respectively. | |
| ***Risk of bias 9**** | | | |
| **Bias** | | **Authors’ judgement** | **Support for judgement** |
| Selection | Representativeness of the exposed cohort | *truly representative of the average ovarian cancer women (describe) in the community | Patients with all FIGO stages of ovarian cancer older than 60 years of age, who underwent standardized surgical treatment at the University Medical Center of the Johannes Gutenberg University Mainz between January 2008 and December 2019, were included |
|  | Selection of the non exposed cohort | *drawn from the same community as the exposed cohort |  |
|  | Ascertainment of exposure | *secure record (e.g., surgical records) | The archives and the electronically patients' records |
|  | Demonstration that outcome of interest was not present at start of study | *yes | Outcomes need to be obtained from follow-up |
| Comparability | Comparability of cohorts on the basis of the design or analysis | *study controls for who had received epidural anesthesia and/or analgesia | Factors were collected according to the current national guidelines, which may influence the postoperative prognosis in OC patients. |
|  |  | *study controls for any additional factor - (These criteria could be modified to indicate specific control for a second important factor.) | The Cox proportional hazards regression model was determined for multivariate analyses of the survival time after debulking  surgery. |
| Outcome | Assessment of outcome | * record linkage | Medical records |
|  | Was follow-up long enough for outcomes to occur | yes | The follow‑up ended at death, and  the longest follow‑up period lasted nearly 11.5 years (June 2008‑December 2019 according to 138 months). |
|  | Adequacy of follow up of cohorts | *description provided of those lost | The loss of follow-up rates were provided for both groups |
