# Supplementary material for: Effects of epidural anesthesia on the prognosis of ovarian cancer—a systematic review and meta-analysis
Source: BMC Anesthesiol. 2023 Nov 29;23:390. doi: 10.1186/s12871-023-02352-1 (PMC10685604; doi:10.1186/s12871-023-02352-1)
Supplement: Supplementary file 2 — Additional file 2: Supplementary Table 2. The summary of the HRs and 95% CI obtained from COX regression models for ovarian cancer patients as reported in the 8 studies-Summary of COX regression analysis. [file 12871_2023_2352_MOESM2_ESM.doc]

| **Summary of Cox Regression Analysis** | | | | | | | | | |  |  |
| --- | --- | --- | --- | --- | --- | --- | --- | --- | --- | --- | --- |
|  | **De Oliveira 2011（TTR）** | **Capmas P 2012(TTR)** | **Elias K.M 2015(TTR)** | **Huang 2018(OS)** | **Lacassie 2013(TTR)** | **L Lin 2011(OS)** | **Tseng 2018 (TTR)** | **Anic 2022(TTR)** | |  |  |
| **Age** |  | Cut-off=53y | 0.993(0.974,1.012) |  |  |  | 1(0.99,1.01) |  | |  |  |
| <50y | 1 |  |  |  |  |  |  |  | |  |  |
| 50-69y | 1.87(1.15,3.04)* | 1.22(0.62,2.40) |  |  |  |  |  |  | |  |  |
| >70y | 2.69(1.40,5.17) * |  |  |  |  |  |  |  | |  |  |
| **ASA physical status** | 1.16(0.74,1.83) |  |  |  |  |  |  |  | |  |  |
| Ⅰ |  |  |  | 1 |  |  |  |  | |  |  |
| Ⅱ |  |  |  | 1.206(0.830,1.752) |  |  |  |  | |  |  |
| Ⅲ |  |  |  | 1.061(1.051,2.439) * |  |  |  |  | |  |  |
| **Anesthesia/analgesia group** | | | | | |  |  |  | |  |  |
| Patient-controlled analgesia | 1 |  |  | 1 | 1 | 1.214(1.075,1.431) * | 1.33(1.07,1.65) * |  | |  |  |
| Epidural analgesia | 0.86(0.52,1.41) | 1.18(0.61,2.31) | 0.736(0.491,1.103) |  | 0.73(0.40,1.33) | 1 |  |  | |  |  |
| Epidural anesthesia plus analgesia | 0.37(0.19,0.73) * |  |  | 0.599(0.419,0.855) * |  |  | 1 |  | |  |  |
| **Blood loss** | 1(1,1) |  | 1(1,1) |  |  |  |  |  | |  |  |
| **Preoperative CA125** | 1(1,1) |  |  | 1.471(1.145,1.890) * |  | 1.107(1.054,1.189) * |  |  | |  |  |
| **Units transfused intraoperative** |  |  | 1.697(0.995,2.894) |  |  |  |  |  | |  |  |
| 0 | 1 |  |  |  |  |  |  |  | |  |  |
| 1-2 | 0.95(0.59,1.53) |  |  |  |  |  |  |  | |  |  |
| ≥3 | 1.26(0.64,2.48) |  |  |  |  |  |  |  | |  |  |
| **Stage** | | | | | | |  |  | |  |  |
| Ⅰ | 1 |  |  | 1 |  | 1 |  |  | |  |  |
| Ⅱ | 0.79(0.32,1.97) |  |  | 1.351(0.337,5.416) |  | 2.197(1.001,5.298) * |  |  | |  |  |
| Ⅲ | 2.63(1.18,5.82) * |  |  | 6.067(1.929,19.075) * |  | 3.276(1.906,6.517) * |  | ＞Ⅱa 2.14(0.38,12.21) | |  |  |
| Ⅲa |  |  | 0.347(0.123,0.976) * |  |  |  |  |  | |  |  |
| Ⅲb |  |  | 0.752(0.388,1.457) |  |  |  | 1(b-c) |  | |  |  |
| Ⅲc |  |  | 1 |  |  |  |  |  | |  |  |
| Ⅳ | 5.03(1.79,14.15) * |  |  | 13.473(4.053,44.782) * |  | 5.432(2.274,12.669) * | 1.13(0.90,1.41) |  | |  |  |
| **Grade** | 0.89(0.46,1.70) | 0.66(0.40,1.06) |  |  |  |  |  |  | |  |  |
| Ⅰ |  |  |  |  |  | 1 |  |  | |  |  |
| Ⅱ |  |  |  |  |  | 1.325(1.152,1.691) * |  | 3.09(1.72,5.55) | |  |  |
| Ⅲ |  |  |  |  |  | 1.360(1.212,1.612) * |  |  | |  |  |
| **Cell type** | | | | | | | | | |  |  |
| Serous | 1 | 3.53(1.42,8.74) * |  |  |  |  |  | |  |  |  |
| Mucinous | 0.62(0.26,1.48) |  |  |  |  |  |  | |  |  |  |
| Mixed müllerian | 0.54(0.20,1.44) |  |  |  |  |  |  | |  |  |  |
| Clear cell | 0.74(0.35,1.58) |  |  |  |  |  |  | |  |  |  |
| Endometrial | 0.71(0.43,1.15) |  |  |  |  |  |  | |  |  |  |
| Undifferentiated | 1.71(0.21,14.04) |  |  |  |  |  |  | |  |  |  |
| Carcinosarcoma |  |  | 4.323(1.950,9.585) * |  |  |  |  | |  |  |  |
| **Operating time** |  | Cut-off=425min  2.77(1.33,5.78) * | 0.880(0.690,1.123) |  |  |  |  | |  |  |  |
| **BMI** |  |  | 1.026(0.991,1.062) |  |  |  |  | |  |  |  |
| **Race** |  |  | 1.534(0.977,2.407) |  |  |  |  | |  |  |  |
| **Volatile anesthetic** | | | | | | |  | |  |  |  |
| Sevoflurane |  |  | 1 |  |  |  |  | |  |  |  |
| Desflurane |  |  | 0.563(0.330,0.962) * |  |  |  |  | |  |  |  |
| **Level of surgical debulking** | | | | | | | | |  |  |  |
| No residual |  |  | 1 |  |  | 1 | 1 | |  |  |  |
| ＜5 mm residual |  |  | 1.779(1.166,2.713) * |  |  |  | 1-10mm 1.71(1.45,2.01) * | | 1.14(0.61,2.12) |  |  |
| 5 mm–1 cm residual |  |  | 3.093(1.727,5.538) * |  |  |  |  | |  |  |  |
| ＜2cm |  |  |  |  |  | 1.221(1.115,1.426) * | ＞10mm 1.99(1.63,2.44)* | |  |  |  |
| ≥2cm |  |  |  |  |  | 2.554(1.297,4.835) * |  | |  |  |  |
| **Lymphatic metastasis** | | | | | | |  | |  |  |  |
| No |  |  |  | 1 |  |  | 1 | |  |  |  |
| Yes |  |  |  | 1.970(1.468,2.642) * |  |  |  | |  |  |  |
| **Lymphatic metastasis** | | | | | | | 0.94(0.72,1.23) | |  |  |  |
| None |  |  |  |  |  | 1 |  | |  |  |  |
| Systematic  lymphadenectomy |  |  |  |  |  | 1.213(1.102,1.476) * |  | |  |  |  |
| Unsystematic  lymphadenectomy |  |  |  |  |  | 1.528(1.053,2.502) * |  | |  |  |  |
| **Bulky upper abdominal disease** |  |  |  |  |  |  | 1.51(1.23,1.86) *  No(reference) | |  |  |  |
| **IP chemotherapy** |  |  |  |  |  |  | 0.63(0.55,0.72) *  No(reference) | |  |  |  |
| *means results had statistical significance | | | | | | | | | |  |  |

**Cox Regression Model Summary**
