# Supplementary material for: Effects of epidural anesthesia on the prognosis of ovarian cancer—a systematic review and meta-analysis
Source: BMC Anesthesiol. 2023 Nov 29;23:390. doi: 10.1186/s12871-023-02352-1 (PMC10685604; doi:10.1186/s12871-023-02352-1)
Supplement: Supplementary file 3 — Additional file 3: Supplementary Table 3. Including patients number of GEA and GA, EIP or EP and adminstered medicine, intravenous analgesia medicine, postoperative pain scores- Detailed comparison of GEA and GA. [file 12871_2023_2352_MOESM3_ESM.rtf]

Detailed comparison of GEA and GA	
Study	GEA:GA
(n:n)	Epidural anesthesia	Intravenous analgesia	Postoperative pain scores 	
Anic 2022	71:39	Intraoperative: bupivacaine 0.25% or 
 ropivacaine 0.375% with or without addition of epidural sufentanil 
Postoperative:bupivacaine 0.125% with or without fentanyl
Mode: patient_controlled

	Medicine: piritramide
Mode:patient-controlled	All patients were cared for by a physician_based acute 
pain service affiliated to the Department of Anesthesiology
	
Campas P 2012	47:47	Intraoperative: /
Postoperative:A bolus of 0.2% ropivacaine 
(20-25 ml) 45 to 60 minutes before the end of surgery. 0.2% ropivacaine and morphine (200ml:5mg), 3days
Mode:3-6ml/h, 3ml, 20mins 	/	/	
Deoliveira 2011	EIP:GA
26:127
EP:GA
29:127	Intraoperative£ºa bupivacaine 10-mg bolus
and an infusion of 12.5 mg/hr
Postoperative:bupivacaine 1 mg/mL plus hydromorphone 10 Kg/mL, 48 to 72 hrs, 
Mode: 4ml/h, 4ml, 12min	Medicine: hydromorphone 
Mode: 0.2-mg bolus with a lockout of
15 mins	/	
Elias 2015	134:60	Intraoperative: /
Postoperative: bupivacaine with or without hydromorphone
Mode: patient_controlled	/	/	
Huang 2018	168:471	Intraoperative: lidocaine
Postoperative: bupivacaine or ropivacine	Medicine:fentanyl	/	
Lacassie 2013	37:43	Intraoperative: 0.1%–0.5% bupivacaine with or without fentanyl
Postoperative: a mixture of local anesthetics and opioids, at least 48hrs
Mode:patient-controlled	Medicine: morphine
tramadol with morphine as rescue analgesic
Mode:patient-controlled	Visual analog scale scores 
<4	
Lin 2011	106:37	Intra-, and postoperative: bupivacaine 0.125% or ropivacaine 0.150% and morphine 6–8 mg, 48h	Medicine: fentanyl
Mode:bolus:10ug, lockout time:5 min	/	
Tseng 2018	435:213	Intra-, and postoperative: 0.05% bupivacaine with or without hydromorphone, fentanyl, or morphine. 
Mode:3ml/h, 3ml, 30min	Medicine:narcotic	All patients were cared for by Acute Pain Service
of the Department of Anesthesiology.	
